# Performance analysis of novel toxin-antidote CRISPR gene drive systems

**DOI:** 10.1186/s12915-020-0761-2

**Published:** 2020-03-12

**Authors:** Jackson Champer, Isabel K. Kim, Samuel E. Champer, Andrew G. Clark, Philipp W. Messer

**Affiliations:** 1 Department of Computational Biology, Cornell University, Ithaca, NY 14853 USA; 2 Department of Molecular Biology and Genetics, Cornell University, Ithaca, NY 14853 USA

**Keywords:** Gene drive, Confined, Modeling, Toxin-antidote, CRISPR, Population modification, Population suppression, Biotechnology

## Abstract

**Background:**

CRISPR gene drive systems allow the rapid spread of a genetic construct throughout a population. Such systems promise novel strategies for the management of vector-borne diseases and invasive species by suppressing a target population or modifying it with a desired trait. However, current homing-type drives have two potential shortcomings. First, they can be thwarted by the rapid evolution of resistance. Second, they lack any mechanism for confinement to a specific target population. In this study, we conduct a comprehensive performance assessment of several new types of CRISPR-based gene drive systems employing toxin-antidote (TA) principles, which should be less prone to resistance and allow for the confinement of drives to a target population due to invasion frequency thresholds.

**Results:**

The underlying principle of the proposed CRISPR toxin-antidote gene drives is to disrupt an essential target gene while also providing rescue by a recoded version of the target as part of the drive allele. Thus, drive alleles tend to remain viable, while wild-type targets are disrupted and often rendered nonviable, thereby increasing the relative frequency of the drive allele. Using individual-based simulations, we show that Toxin-Antidote Recessive Embryo (TARE) drives targeting an haplosufficient but essential gene (lethal when both copies are disrupted) can enable the design of robust, regionally confined population modification strategies with high flexibility in choosing promoters and targets. Toxin-Antidote Dominant Embryo (TADE) drives require a haplolethal target gene and a germline-restricted promoter, but they could permit faster regional population modification and even regionally confined population suppression. Toxin-Antidote Dominant Sperm (TADS) drives can be used for population modification or suppression. These drives are expected to spread rapidly and could employ a variety of promoters, but unlike TARE and TADE, they would not be regionally confined and also require highly specific target genes.

**Conclusions:**

Overall, our results suggest that CRISPR-based TA gene drives provide promising candidates for flexible ecological engineering strategies in a variety of organisms.

## Background

A successful gene drive can rapidly spread through a population by biasing inheritance in favor of the drive allele [1–7]. This can be used for population modification, or, with an appropriate drive arrangement, population suppression [1–7]. The potential applications of such drives are numerous, with perhaps the most promising involving the modification or suppression of mosquito populations to prevent transmission of vector-borne diseases such as malaria or dengue [1–3, 5]. Several possible “payload” (carried by the drive) genes that could prevent the transmission of vector-borne disease already exist [8, 9]. Similar techniques can potentially be used against invasive species or agricultural pests. However, major obstacles must still be overcome before gene drives could fulfill their promise.

Homing drives based on CRISPR-Cas9 are the best-studied form of gene drive and have been constructed in yeast [10–13], flies [14–20], mosquitoes [21–23], and mice [24]. These constructs work by cleaving a wild-type allele at the guide RNA (gRNA) target site and then copying the drive allele into that site during homology-directed repair, a natural cell process. If cleavage is repaired by end-joining, also part of natural DNA repair in the cell, mutations can be created at the target site [17]. This often results in the formation of a resistance allele, since the mutated target site is no longer a match to the drive’s gRNA. This prevents future cleavage by the drive and thus impairs its spread. Such resistance alleles can form in the germline as an alternative to homology-directed repair as well as during early embryo development by maternally deposited Cas9 and gRNAs [17]. Some strategies for reducing resistance allele formation have already been successfully tested, including gRNA multiplexing [18], improved promoters [18, 25], and selection of a highly conserved target site where mutations are not tolerated [26]. This latter method, when combined with an improved promoter, recently resulted in the successful suppression of *Anopheles gambiae* in experimental cage populations [26].

While very promising for suppression drives, such a strategy may be difficult to apply to population modification drives. This is because it relies on the principle that resistance alleles render the target gene nonfunctional, thereby enabling them to contribute to the overall goal of population suppression even if they slow the spread of the drive allele. A population modification drive, by contrast, would only be able to remain viable while removing resistance alleles if the drive targets an essential gene and itself contains a recoded version of the target gene that restores its function. This would require targeting a site that can be sufficiently recoded without rendering the target gene nonfunctional (i.e., the target sequence is not fully constrained). Yet at such a site, it should then also be possible for resistance alleles to maintain gene function [27].

A homing drive with two gRNAs targeting a haplolethal gene was able to spread through a cage population in a recent study [28], but this type of drive may be vulnerable to formation of resistance alleles when rare incomplete homology-directed repair events lead to copying of the recoded region but not the “payload” gene, essentially forming a functional resistance allele. Aside from this limitation, all homing drives also require Cas9 cleavage to occur specifically in the germline in order to allow for homology-directed repair instead of end-joining, which tends to predominate at other stages (particularly when cleavage occurs in the embryo). This requires choosing a suitable promoter, which may often be difficult to find in non-model species and could thereby prove to be a barrier to development of homing drives.

Another inherent feature of homing drives that could limit their utility is the propensity to spread to distant populations with even small levels of migration [29], making it difficult to confine such a drive to a specific geographic region. This could be particularly undesirable for applications where the goal is suppression of invasive species or agricultural pests outside their native range [30]. Thus, new gene drive options are needed that are effective and flexible and can be confined to a target region.

One possible strategy for developing an efficient drive is to avoid the need for homology-directed repair by using a toxin-antidote (TA) drive system. This mechanism is often seen in natural drives [31] and has been successfully applied for the *Medea* system [32]. However, *Medea* elements proved to be highly specific to *Drosophila* and difficult to transfer into other species. Other proposals for TA systems [33–38] have similar difficulties in implementation. CRISPR nucleases could in principle be used to create highly flexible systems, where the “toxin” would consist of Cas9 and gRNAs targeting an essential gene. The “antidote” would be a copy of the gene carried by the drive that has been recoded to render it immune to cleavage. With both the toxin and antidote as part of the drive allele, it can steadily convert wild-type alleles to disrupted alleles in the population. These will then systematically be removed from the population by natural selection, thereby increasing the relative frequency of the drive over time. Two implementations of such systems have recently been demonstrated in *Drosophila*, and both were able to successfully spread through cage populations within just a few generations without any apparent evolution of resistance [39, 40]. With even small fitness costs, some of these CRISPR TA designs have the potential to be regionally confined to a target population.

A variety of TA systems are conceivable, depending on the nature of the target gene and the intended application of the drive (population modification or suppression). In this study, we provide a detailed discussion of several such systems, including those based on haplolethal genes and genes that are essential but haplosufficient (where disrupted alleles are recessive lethal), as well as specific genes required for sperm development.

## Results

### General TA drive principles and systems

The underlying design principle of all TA drive systems is that the drive alleles contain a toxin together with an antidote that rescues the effect of the toxin. We assume that the toxin is a CRISPR nuclease targeting an essential gene that will be disrupted and rendered nonfunctional when mutations are introduced at the cut sites through end-joining or homology-directed repair. The antidote consists of a recoded version of the gene, which does not match the gRNAs and therefore cannot be cleaved by the drive. Cells or individuals exposed to the toxin will often be nonviable, unless rescued by a drive allele. In contrast to homing drives that spread by directly increasing the number of drive alleles, TA drives spread by reducing the number of wild-type alleles (and thus still increasing the relative frequency of the drive). Various potential arrangements and targets for TA systems can be conceived. In this study, we will focus on three general classes of such systems:

- TARE (Toxin-Antidote Recessive Embryo). These drives target an essential but haplosufficient gene. Disrupted alleles are recessive lethal (i.e., one functional copy of the gene is required for viability, which can be a drive or wild-type allele).
- TADE (Toxin-Antidote Dominant Embryo). These drives target a haplolethal gene (i.e., two functional copies of the gene are required for viability).
- TADS (Toxin-Antidote Dominant Sperm). These drives target a gene that is transcribed in gametocytes after meiosis I in males, with this expression being critical for successful spermatogenesis. We assume that all sperm with a disrupted target allele are nonviable.

**Fig. 1:**
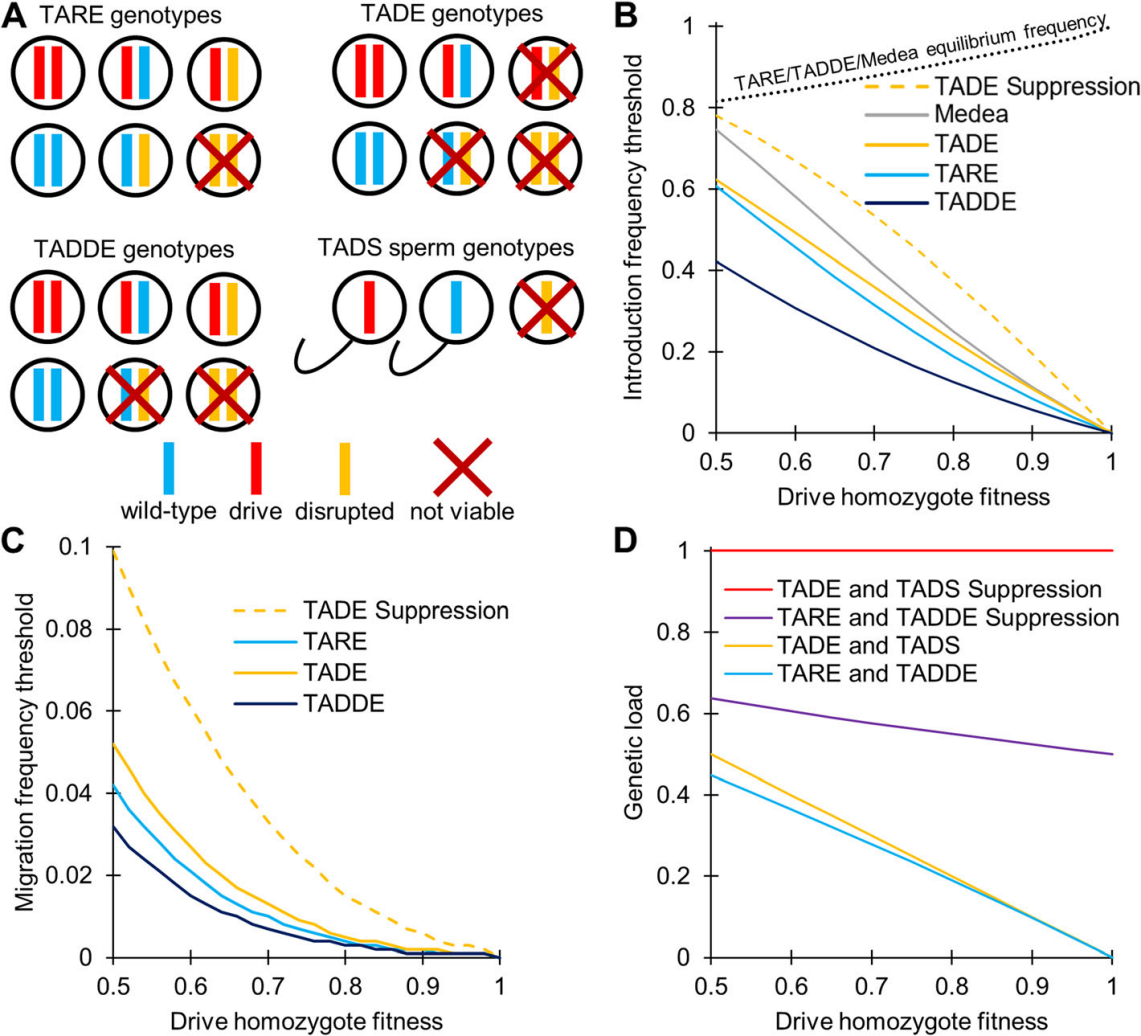
Overview of TA systems and performance characteristics. **a** Illustration of viable and nonviable genotypes for the different types of TA systems. **b** TARE, TADE, TADDE, and *Medea* drives have fitness-dependent introduction thresholds, above which the drive will increase in frequency and below which it will decrease. Frequencies represent the introduction of drive heterozygotes (with “ideal” drives in the deterministic model). The black dotted line shows the final drive allele equilibrium frequency for TARE, TADDE, and *Medea*, though all individuals should carry at least one copy of the drive at these equilibria. **c** Migration thresholds for the different TA systems, showing the per generation influx of new migrants (homozygotes for TARE, TADE, and TADDE, heterozygotes for TADE suppression) as a fraction of the population that is required for the drive to eventually spread to all individuals in the population (below this level, the drive reaches a low equilibrium frequency). Note that these thresholds likely overestimate the invasion potential of a TADE suppression system, since suppression and subsequent reduction in migration will occur in distant populations that send migrants as the drive increases in frequency. **d** The genetic load imposed by idealized TA drives as a function of the drive homozygote fitness in the deterministic model. Eradication will only occur if the genetic load can overcome the fitness advantage of individuals at low population density. Note that “TARE and TADDE suppression drive” refers to a distant-site TARE or TADDE drive located in a female fertility gene (as in a TADE suppression drive). Such a drive reaches a moderate equilibrium frequency and is thus unable to impose a large genetic load on the population like a TADE suppression drive

Figure 1a shows which genotypes are rendered nonviable in each of these classes of drive. The detailed features of these systems will be discussed in the relevant sections below. Generally, TARE systems are aimed for population modification while TADE and TADS systems can be used for both population modification and suppression. For TADE or TADS suppression, we assume that the drive and target loci are unlinked and that the drive is placed in an essential but haplosufficient fertility gene (that only affects fertility in one sex for TADE and specifically males for TADS), disrupting the gene with its presence (not by targeting it with gRNAs) so that drive homozygotes of one sex are sterile. We further discuss a population modification variant of TADE that we term Toxin-Antidote Double Dominant Embryo (TADDE) drive, which still targets a haplolethal gene but has a stronger rescue element such that a single drive allele is sufficient for an individual to be viable (Fig. 1a), allowing the drive to spread faster with a lower invasion threshold than TADE drive. Finally, we will discuss a variant of TADS where the drive is located on the Y chromosome (termed TADS Y-suppression). Such a system is expected to exhibit similar dynamics to previously studied X-shredder drives [41–43].

### Overview of population dynamics

Before conducting a full analysis of each individual drive, we will provide an overview of their expected population dynamics, as compared to a homing drive, X-shredder, and *Medea* system. For these initial analyses, we assume “ideal” drives (no resistance evolution, 100% target cutting activity in the germline for the homing and TADE drives and both germline and early embryo cutting for TARE, TADDE, and TADS). For *Medea*, we assume that all offspring of mothers with a *Medea* allele will be nonviable unless they receive a *Medea* allele from either parent. We further assume a panmictic population of infinite size. This allows us to use a deterministic model, specified by recursion equations for the expected changes in genotype frequencies between discrete generations (see the “Methods” section).

All of the TA systems we tested are able to spread quickly through the population in this idealized model (Figure S1), but most have frequency thresholds below which the drive will not invade if it carries a fitness cost (Fig. 1b). Realistic drives should carry at least a small fitness costs from drive components itself (from expression of the CRISPR nuclease, for example), and payload genes would likely add additional fitness costs (although these might be removed if mutations render the payload nonfunctional). When introduced above the threshold frequency, the drive is expected to increase in frequency and spread successfully, while below the frequency, it would likely be eliminated from the population. In contrast, TADS drives, as well as homing drives [4, 44] and X-shredders [44], all have a zero-threshold introduction frequency unless fitness costs are very high (drive homozygote fitness < 0.5 in idealized forms). These drives would therefore be expected to spread from any release frequency if they are expected to spread at all. Note, though, that there exists a narrow range of fitness values under which homing [4, 44] and TADS drives also have a nonzero introduction frequency threshold.

The presence of an introduction threshold can allow a drive to be confined to a target population if the migration rate into a connected population is below a “migration threshold.” Note that this migration threshold is different from the “introduction threshold” because migrants could accumulate over time to eventually exceed the introduction threshold [45–50]. However, if an introduction threshold exists, a migration threshold will exist as well. To assess the migration thresholds for our drives in a simple scenario, we studied a wild-type population experiencing a fixed rate of immigration from drive-carrying individuals each generation (presumably from a separate population where the drive is already established). The migration threshold then represents the minimum rate of immigration (as a fraction of the population) needed for the drive to eventually spread through the population (Fig. 1c). These migration thresholds follow the same pattern as the introduction thresholds, though all are lower. Note that such thresholds are also representative of the level of effort needed in a continual release strategy, rather than the single-release strategy considered elsewhere in this manuscript.

TARE, TADDE, and *Medea* drives are not expected to go to fixation but instead reach equilibrium frequencies that are dependent on fitness costs (Fig. 1b). At equilibrium, all individuals are expected to carry at least one copy of the drive (Figure S1B), but some will carry disrupted alleles as well. Suppression forms of the drives are potentially capable of inducing high genetic loads (defined as the average net fitness reduction relative to a wild-type population of the same size after the drive reaches an equilibrium), though fitness costs can allow modification-type drives to induce a modest genetic load as well (Fig. 1d). However, loads based on such fitness costs in modification drives will usually be insufficient to eradicate a population or even substantially reduce its numbers, depending on ecological characteristics.

We will next study the individual TA systems more closely, exploring how their dynamics change as drive parameters are varied from the idealized model. The following analyses no longer assume a deterministic model of an infinite population as used in Fig. 1. Instead, they are based on our individual-based simulations, which seek to model a more realistic population of finite size with density regulation. These simulations therefore take stochastic effects into account, which can become particularly relevant for suppression approaches as population size decreases.

### TARE drive

**Fig. 2:**
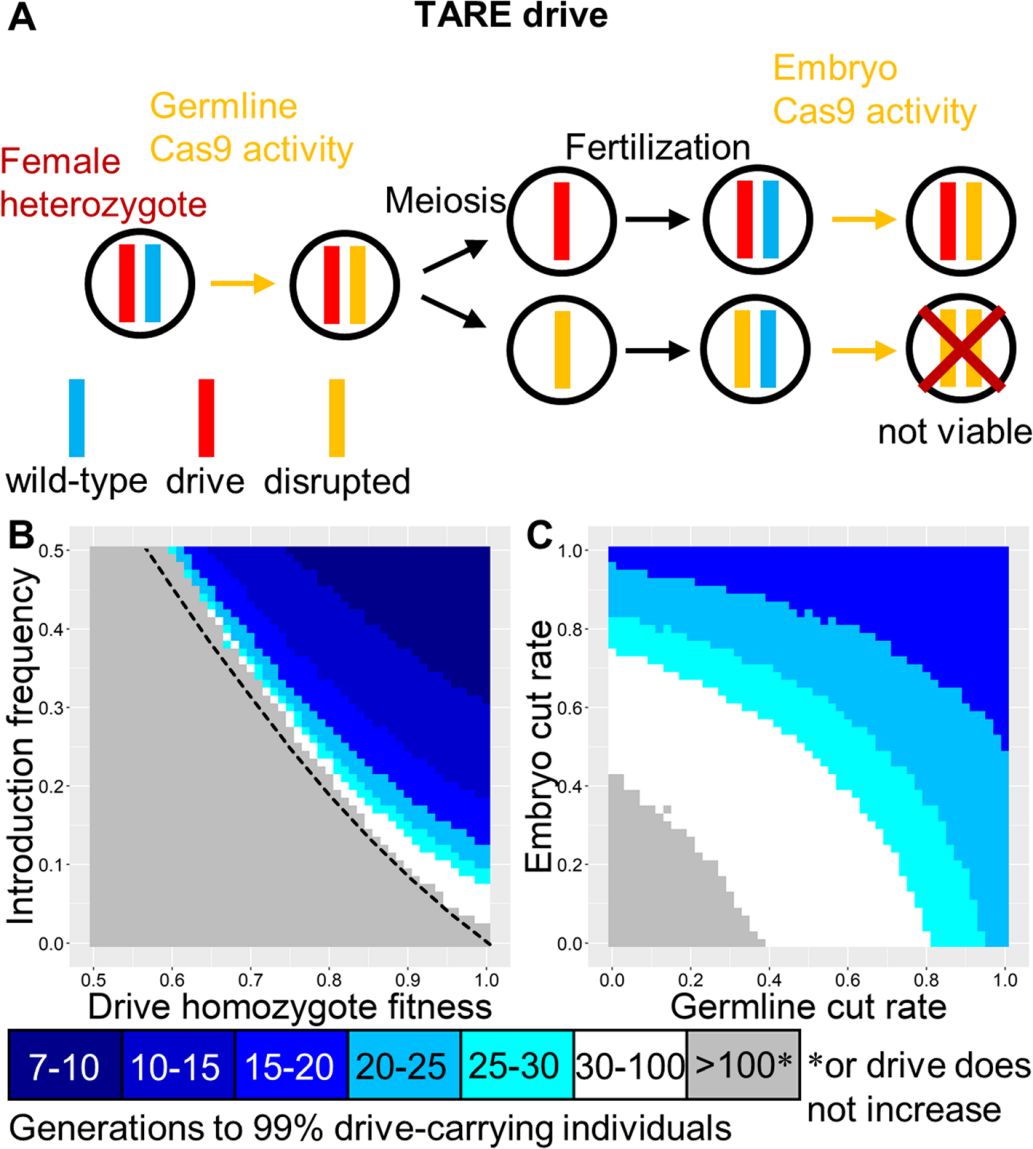
TARE drive. **a** In the TARE drive, germline activity disrupts the target gene, followed by embryo activity in the progeny of drive-carrying females. The target gene is assumed to be essential and haplosufficient, so any individuals inheriting two disrupted (recessive lethal) target genes are nonviable. By contrast, all individuals with at least one wild-type or drive allele are assumed to be viable. **b** The speed at which a TARE drive is expected to reach 99% of individuals in the population with varying introduction frequency and drive fitness. The dashed line indicates the introduction frequency threshold in the deterministic model. **c** Same as **b**, but with varying germline and embryo cleavage rates. Gray means that the drive failed to reach 99% because it spread too slowly or was not able to spread at all

These drives constitute modification drives that target a gene that is essential and haplosufficient (disrupted alleles are recessive lethal), with the drive providing rescue (Fig. 2a). One consequence of this mechanism is that TARE drive will have threshold-dependent invasion dynamics (Figs. 1b and 2b). Another consequence is that embryo Cas9 cleavage from maternally deposited Cas9, which poses a major problem for homing-type drives, actually makes a TARE drive more efficient (Fig. 2c). For example, when a heterozygous female mates with a wild-type male, most of their offspring will end up carrying the drive. This is because those that did not inherit a drive allele from their mother will likely inherit a disrupted target allele, and the wild-type allele inherited from the father will then become disrupted due to maternal Cas9 activity, rendering those individuals nonviable. TARE drives should therefore be highly tolerant of variation in expression from the nuclease promoter. Indeed, the promoter of a TARE drive need not even be restricted to expression in the germline and early embryo. Constitutively active promoters would presumably work equally well (though they may have a higher fitness cost), as long as there is expression in germline or germline precursor cells.

The TARE drive can be “same-site” as in Fig. 2a or a “distant-site” drive in which the drive allele is not located at the same genomic site as the target allele (Figure S2A) (note that Fig. 1a shows genotypes for “same-site” drives). Successful same-site [39] and distant-site (called ClvR) [40] systems have already been engineered with high germline and embryo cut rates and little to no observable fitness costs. Same-site and distant-site systems should have nearly equivalent performance when cut rates are high (Figure S2B and the ClvR study [40]), but the distant-site drive retains higher performance when both the germline and embryo cut rates are low (Figure S3C) since it often has two wild-type alleles available to cleave in this parameter space, rather than one as for the same-site drive. On the other hand, a same-site drive may be easier to engineer since the recoded region is smaller and the natural target gene promoter would drive expression of the rescue allele. The natural promoter and genomic site of the rescue element may also avoid the pitfall of incomplete rescue that is a more significant consideration for distant-site drives.

In our model, TARE systems reach all individuals quickly with a modest release size (Figure S1B), but their rate of increase becomes slowed at high frequencies (Figure S1A), which could be an issue for a population modification strategy where the payload is substantially more effective in homozygotes than heterozygotes. To avoid this, the target of a TARE system could be located on the X chromosome, so that males with only one copy of the disrupted target gene are nonviable (Figure S3D). This would allow the drive allele to fix substantially more quickly than autosomal TARE systems (Figure S3E). However, X-linked TARE drives would not have any cleavage activity in the germline of males and therefore have a slower rate of spread than autosomal systems (Figure S3F), at least until the drive has reached most individuals.

### TADE drive

**Fig. 3:**
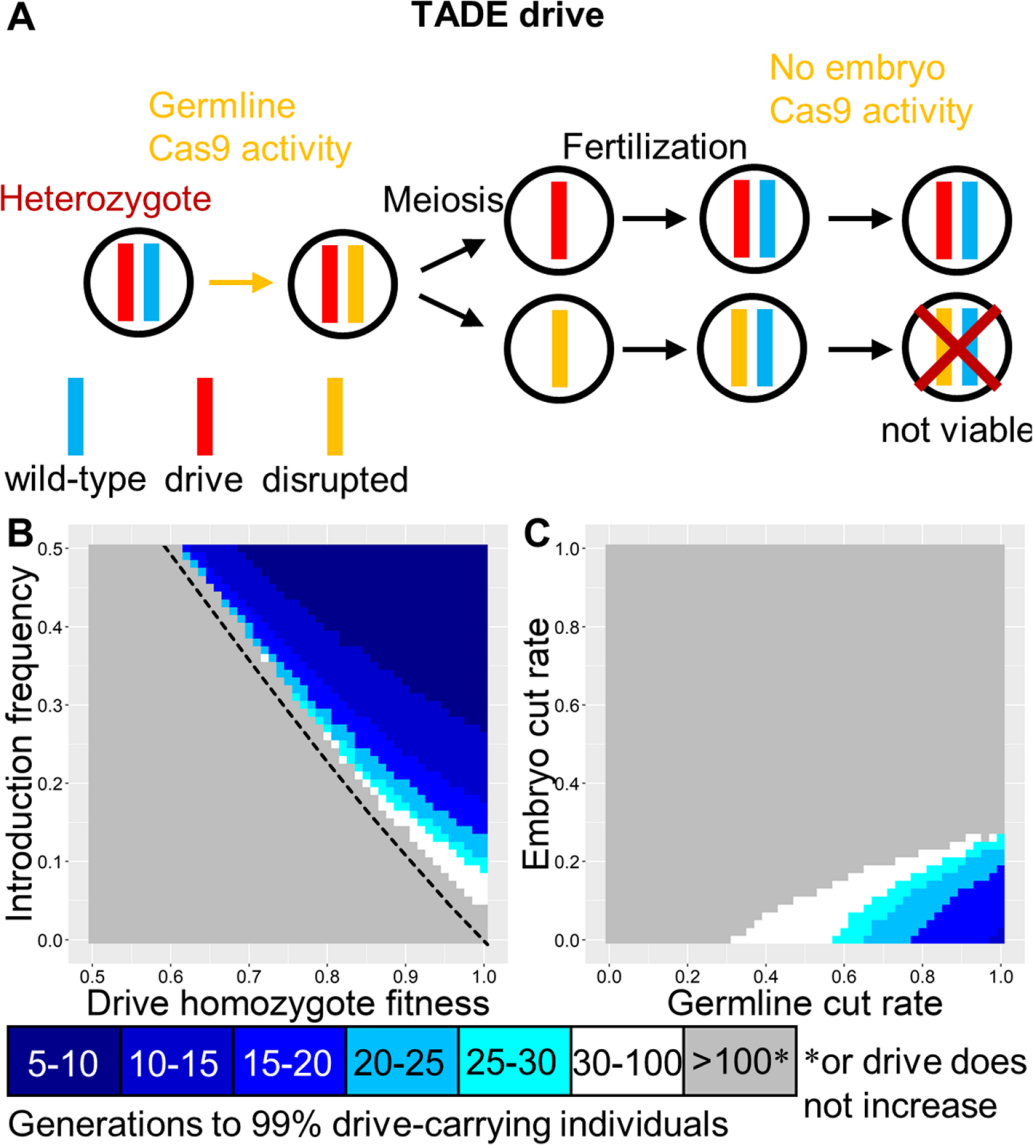
TADE drive. **a** In the TADE drive, germline activity disrupts the target gene, and the nuclease promoter is selected to minimize embryo activity. The target gene is assumed to be haplolethal, so any individuals inheriting one disrupted target allele will be nonviable, even if the other allele is drive or wild-type. **b** The speed at which a TADE drive is expected to reach 99% of individuals in the population with varying introduction frequency and drive fitness. The dashed line indicates the introduction frequency threshold in the deterministic model. **c** Same as **b**, but with varying germline and embryo cleavage rate

These drives target a haplolethal gene, with the drive allele providing rescue (Fig. 3a). Like TARE, such a drive is expected to show threshold-dependent dynamics (Figs. 1b and 3b). However, nuclease cleavage should occur only in germline gametocytes for a TADE drive, rather than in both germline and early embryo. Otherwise, drive/wild-type heterozygotes will not have two functioning copies of the haplolethal gene in all cells, which will likely result in low fitness or death, depending on the magnitude of expression outside the germline. Similarly, embryo cleavage activity would render some drive-carrying individuals nonviable. Though the nuclease promoter should be germline-restricted, it could still have expression before or after the narrow window for homology-directed repair in early meiosis, allowing TADE drives to be somewhat more flexible for promoters than homing drives. With a suitable promoter, the offspring of both males and females that fail to inherit the drive will perish. This allows the TADE drive to spread more rapidly than the TARE drive and quickly fix (Figure S1A). However, substantial embryo resistance would likely thwart such a drive (Fig. 3c).

As with the TARE drive, a TADE drive can be same-site or distant-site (Figure S3A). Both configurations are expected to have similar performance (Figure S3B), but the distant-site drive may remain viable for higher embryo resistance rates when germline cleavage is low (Figure S3C). This is because a low rate of embryo cleavage can help remove wild-type alleles that were not cleaved in the germline due to low germline cleavage rates. The drive alleles in this situation should still remain viable in most instances, since the other wild-type target allele would often remain undisrupted.

### TADE suppression drive

**Fig. 4:**
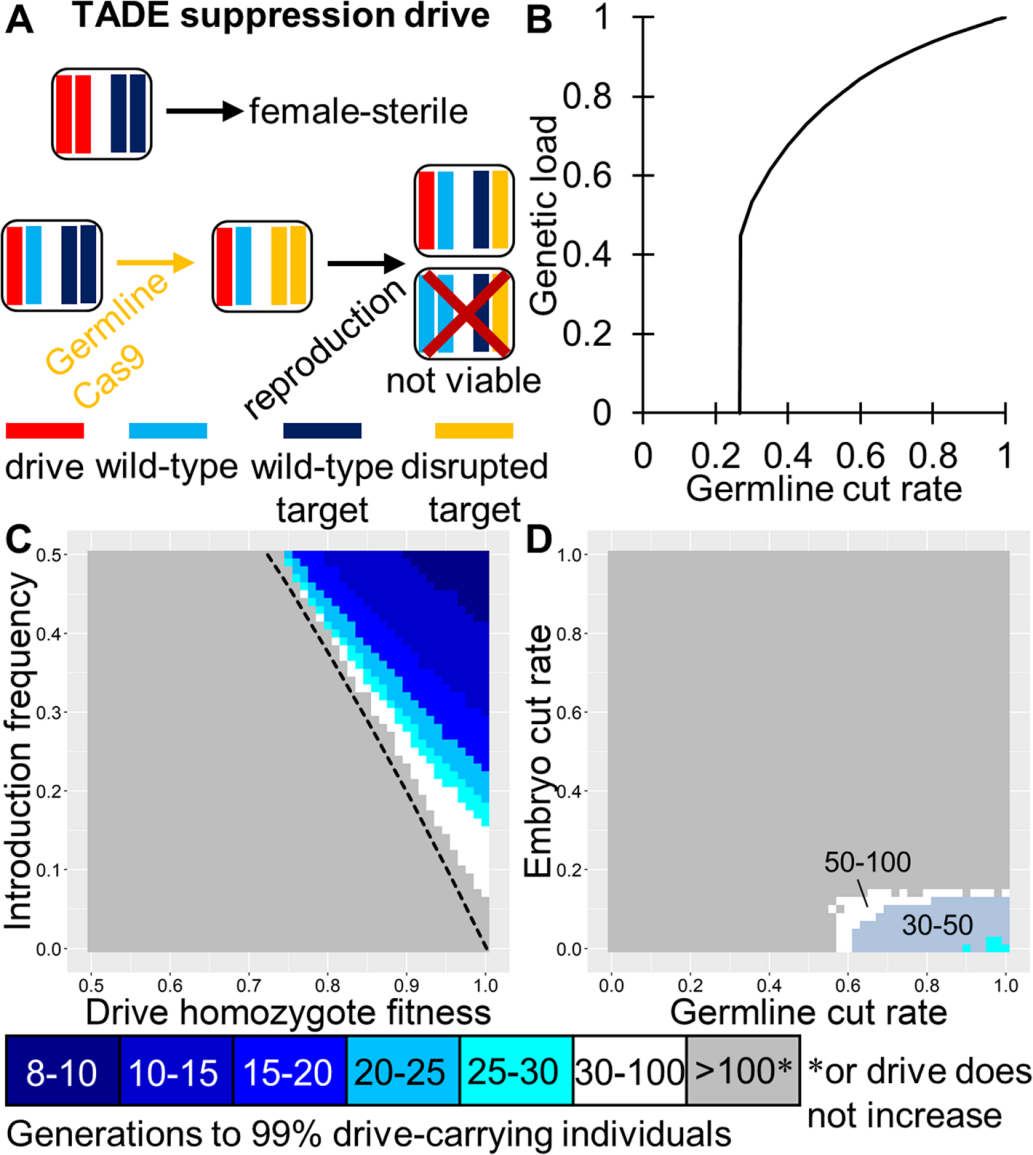
TADE suppression drive. **a** The target gene of a TADE suppression drive is at a different site from the drive allele (modeled as an unlinked site), which is located in a female (or male) fertility gene. The drive disrupts the fertility gene, so female drive homozygotes are sterile (“drive homozygote fitness” does not apply). Germline activity disrupts the target gene, and the nuclease promoter is selected to minimize embryo activity. The target gene is haplolethal, so any individuals inheriting fewer than two wild-type target alleles and/or drive alleles are nonviable. **b** The genetic load imposed by a TADE suppression drive in our deterministic model. If the germline cleavage rate is 100%, eradication will occur. Otherwise, eradication will only occur if the genetic load can overcome the fitness advantage of individuals at low population density. Note that this drive loses the ability to increase in frequency in any population when the germline cut rate is very low. **c** The speed at which the TADE suppression drive reaches 99% of individuals in the population with varying introduction frequency and drive fitness. Full suppression or an equilibrium state will be attained within a few generations of this point. The dashed line indicates the introduction frequency threshold in the deterministic model. **d** Same as **c**, but with varying germline and embryo cleavage rate

The TADE suppression drive is a form of distant-site TADE in which the drive is located in an essential but haplosufficient female (or male, but not both) fertility (or viability) gene, disrupting the gene with its presence (Fig. 4a). Thus, female drive homozygotes are sterile. If the germline cleavage rate is less than 100%, this drive would not fix but instead impose a genetic load on the population (Fig. 4b), defined as the average net fitness reduction relative to a wild-type population of the same size after the drive reaches its maximum frequency. This includes direct fitness effects of the drive regardless of genotype, drive-induced sterility in certain drive homozygotes, and loss of offspring due to nonviable genotypes formed by the drive. In our stochastic model with density regulation, complete eradication is expected to occur when the genetic load is equal to or greater than 1—(1/population growth rate at low density), though eradication may occur before this point due to stochastic effects or if Allee effects begin to contribute to suppression [51]. For the germline cut rates observed experimentally in mosquito and *Drosophila* systems [14–23], this would likely be sufficient to cause complete population eradication. High genetic loads are also possible even if the target gene shows only partial haploinsufficiency (Figure S4, defined as the fitness cost to individuals with a single functioning copy of the target gene). Note that unlike homing drives and X-shredders, TADE suppression drives are expected to show threshold-dependent dynamics (Figs. 1b and 4c), making them regionally confinable systems. In an effective TADE suppression drive, the parameter space for embryo and germline cut rates is even more restricted than for a TADE drive (Fig. 4d), though still within the range demonstrated in mosquito drives [26]. Note that if a TARE drive was similarly placed in a female fertility gene, it would likely lack the power to eradicate the population and only be able to induce a modest genetic load (Fig. 1d).

### TADDE drive

**Fig. 5:**
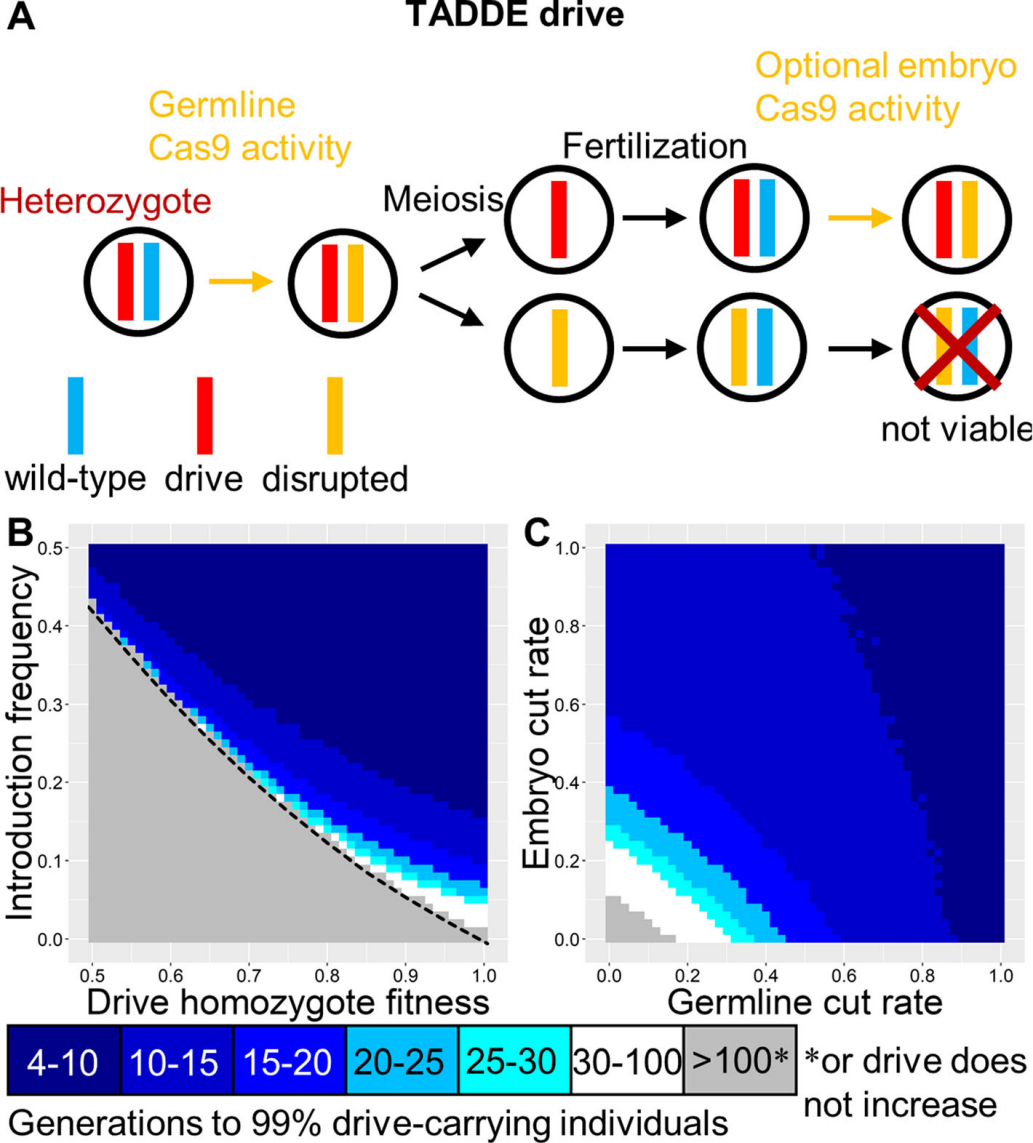
TADDE drive. **a** In the TADDE drive, germline activity disrupts the target gene, and embryo activity is optional (and preferred unless germline cleavage is 100%). The target gene is haplolethal, so any individuals inheriting at least one disrupted target allele are nonviable unless they also inherit a drive allele, which encodes two copies of the gene or provides sufficient expression of the target gene such that only one copy is needed for rescue. **b** The speed at which the TADDE drive is expected to reach 99% of individuals in the population with varying introduction frequency and drive fitness. The dashed line indicates the introduction frequency threshold in the deterministic model. **c** Same as **b**, but with varying germline and embryo cleavage rate

TADDE drives are simply TADE drives in which the rescue element either has two recoded copies of the haplolethal gene or a sufficiently altered promoter to increase expression of the rescue element, such that a single drive allele is sufficient to provide rescue even if paired with a disrupted allele (Fig. 5a). TADDE drives thus allow for the removal of wild-type alleles immediately after disruption in both males and females, while preventing removal of drive-carrying individuals, which occurs in TADE drive offspring when two drive heterozygotes mate. This allows a TADDE drive to spread more quickly (Fig. 2b) with a lower threshold (Fig. 1b) than similarly efficient TADE or TARE systems, while retaining similar threshold-based dynamics (Fig. 5b). Because drive alleles are not automatically removed when paired with disrupted targets, embryo cleavage can be fully tolerated (as well as somatic expression, like in TARE), even though it would not significantly increase the rate of spread of this drive when germline cleavage is already high (Fig. 5c). Same-site and distant-site TADDE drives are expected to have very similar performance except when both germline and embryo cleavage rates are very low (Figure S5).

### TADS drive

**Fig. 6:**
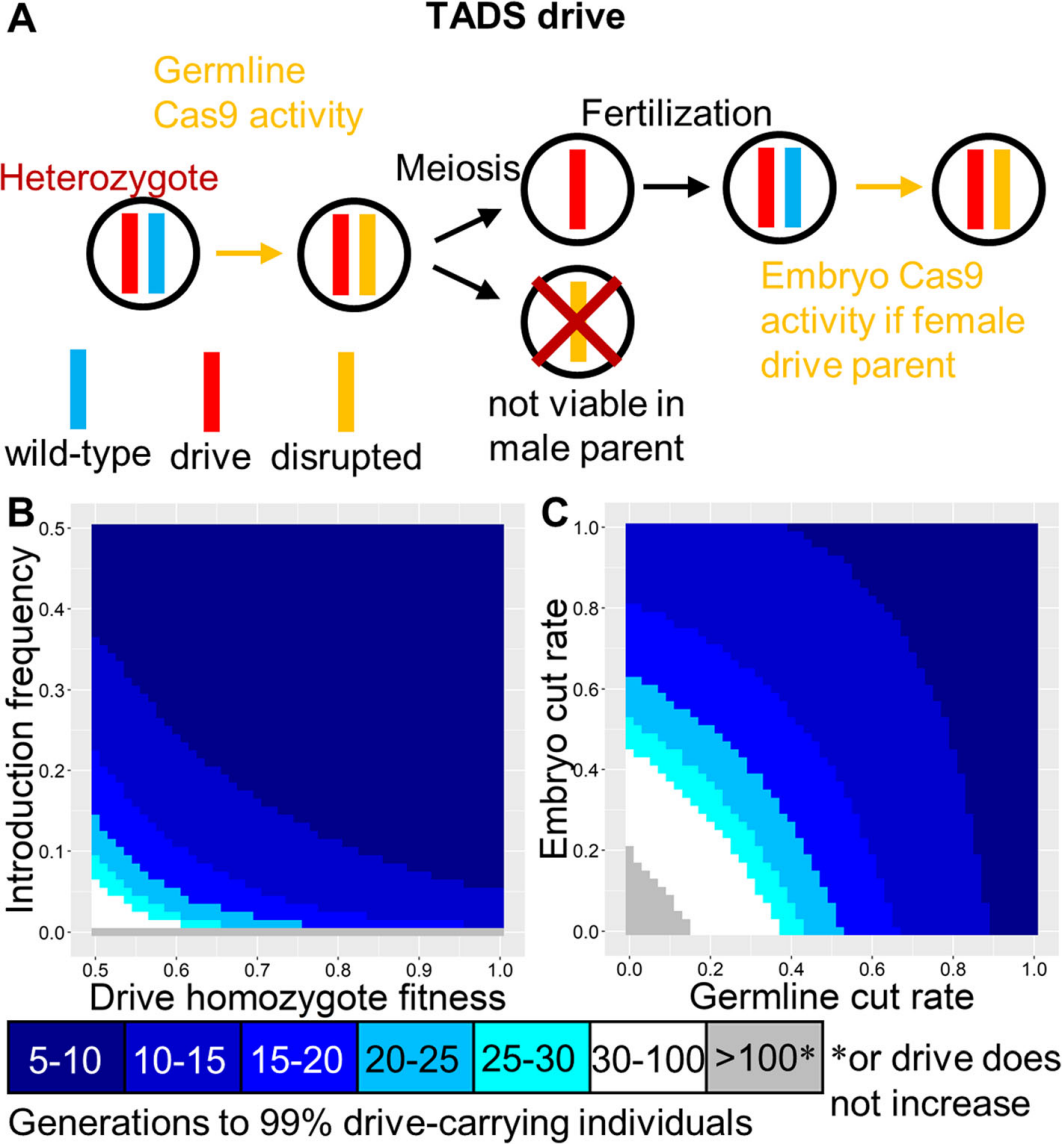
TADS drive. **a** In the TADS drive, germline activity disrupts the target gene, followed by embryo activity in the progeny of drive-carrying females. The target gene is expressed in male gametocytes after meiosis I, and such expression is necessary for development of a viable sperm. Thus, sperm with a disrupted allele are nonviable, and only sperm with a wild-type or drive allele are viable. **b** The speed at which the TADS drive is expected to reach 99% of individuals in the population with varying introduction frequency and drive fitness. **c** Same as **b**, but with varying germline and embryo cleavage rate

These drives target a gene that is transcribed in male gametocytes after meiosis I, and this expression must be critical for successful spermatogenesis such that sperm with a disrupted target allele are nonviable. Thus, only sperm with drive or wild-type alleles can successfully fertilize eggs (Fig. 6a), resulting in rapid spread of the drive (Figure S1A). However, the rate of spread would be somewhat reduced if females can mate with multiple males and sperm could be competing to fertilize eggs. The mechanism by which such a drive spreads is similar to a homing drive, and it therefore has a zero-threshold introduction frequency (Fig. 6b) unless fitness costs are very high, meaning that it would not be expected to remain regionally confined. Somatic expression would likely be fully tolerated for such drives, and they should also allow for a wide variety of promoters varying in both germline and embryo cut rates (Fig. 6c). Nevertheless, finding a suitable target gene could be difficult. Distant-site and same-site configurations of TADS drives should be similar (Figure S6A), although as with the other types of drive, distant-site TADS should perform somewhat better than same-site TADS when both germline and embryo cleavage rates are very low (Figure S6B-C).

### TADS suppression drive

**Fig. 7:**
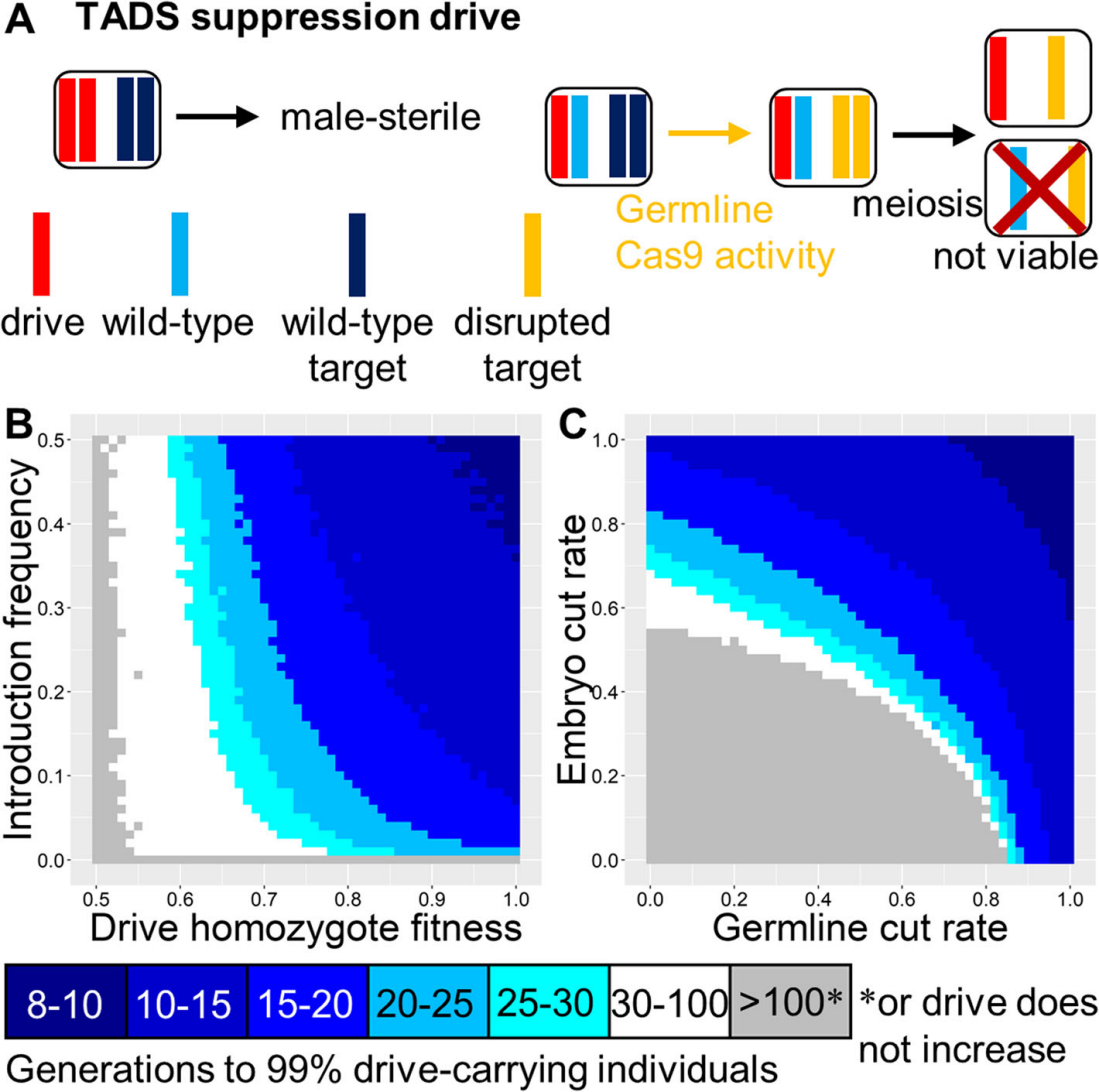
TADS suppression drive. **a** The TADS suppression drive is distant-site, located in a male fertility gene and modeled here with a target gene that is unlinked from the drive allele. Male drive homozygotes are thus sterile (“drive homozygote fitness” does not apply). Germline activity disrupts the target gene, followed by embryo activity in the progeny of drive-carrying females. The target gene is expressed in male gametocytes after meiosis I, and such expression is necessary for the development of a viable sperm. Thus, sperm with a disrupted target allele are nonviable unless they also have a drive allele. **b** The speed at which the TADS suppression drive is expected to reach 99% of individuals in the population with varying introduction frequency and drive fitness. Full suppression would occur within a few generations of this point. **c** Same as **b**, but with varying germline and embryo cleavage rate

A distant-site TADS drive can be configured for population suppression by placing it in an essential but haplosufficient male fertility (or viability) gene, disrupting the gene with its presence (Fig. 7a). Thus, male drive homozygotes would be sterile. Note that because the drive works during spermatogenesis, it would be unable to provide any substantial suppression if located in a female (or both-sex) fertility or viability gene. However, in a male fertility gene, it would be expected to cause complete population eradication with a zero-threshold invasion frequency (Fig. 7b) unless fitness costs are very high, similar to homing drives targeting a fertility or viability gene or to X-shredders (Figure S1A). The suppression form of TADS should be less tolerant of low embryo and germline cut rates than modification TADS, but such drives can still achieve success over a wide range of values (Fig. 7c).

### TADS Y-linked suppression drive

**Fig. 8:**
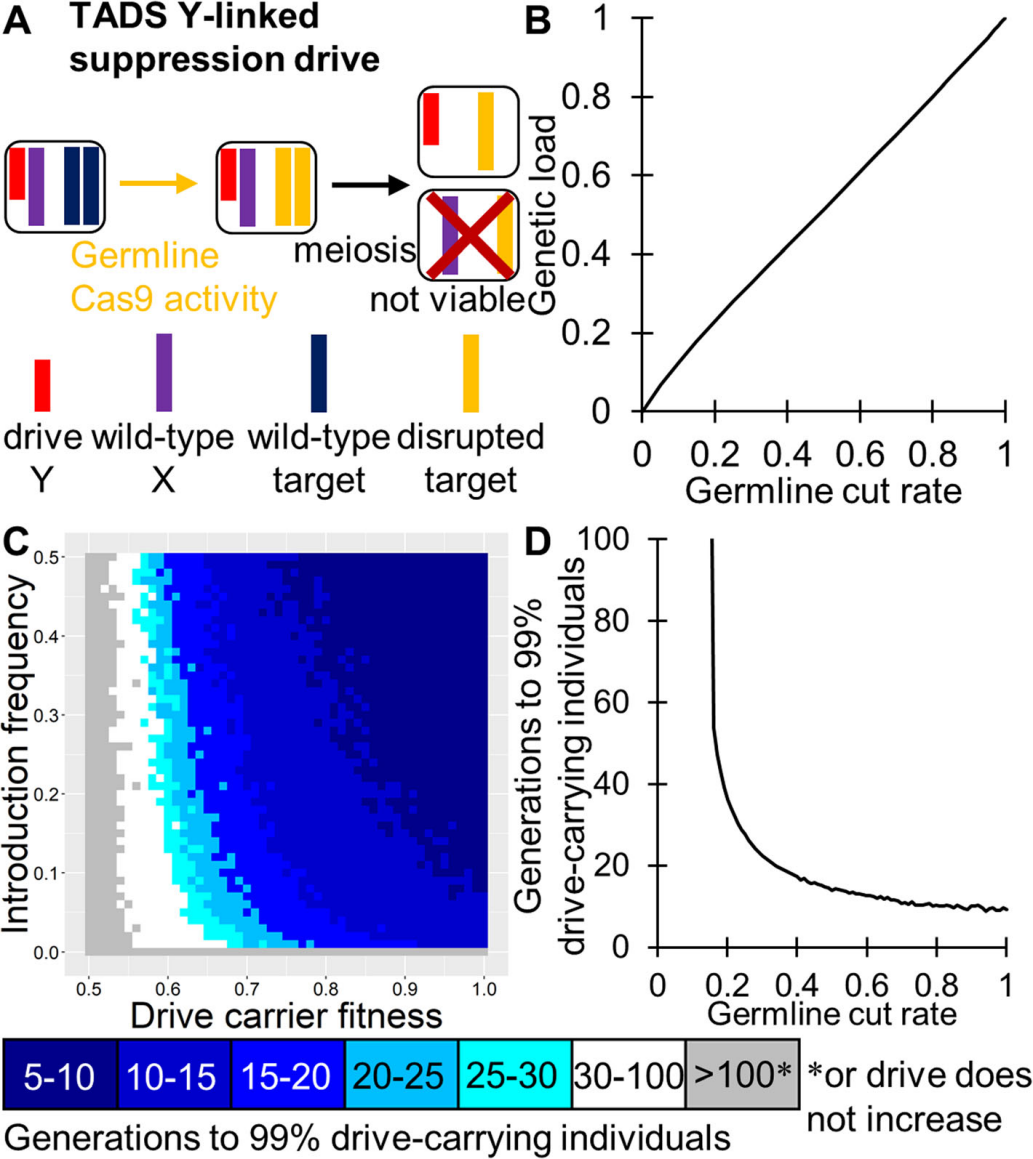
TADS Y-linked suppression drive. **a** The TADS Y-linked suppression drive is distant-site. It is located on the Y chromosome and has a target gene that is not linked to the drive allele (modeled here to be on an autosomal chromosome). Germline activity disrupts the target gene, followed by embryo activity in the progeny of drive-carrying females. The target gene has expression in male gametocytes after meiosis I, and such expression is necessary for development of a viable sperm. Thus, sperm with a disrupted target allele are nonviable unless they also have a drive allele. **b** The ability of the TADS Y-linked suppression drive to suppress a population in our deterministic model. If the germline cleavage rate is 100%, suppression will occur. Otherwise, suppression will occur only if the genetic load can overcome the fitness advantage of individuals at low population density. **c** The speed at which the TADS suppression drive is expected to reach 99% of individuals in the population with varying introduction frequency and drive fitness. Full suppression or an equilibrium state will be attained within a few generations of this point. **d** Same as **c**, but with varying germline cleavage rate (the Y-linked drive can only be carried by males, so there would likely not be any embryo cleavage—however, if there was paternal activity due to unusually high nuclease/gRNA expression or stability, this would be expected to further increase drive efficiency)

**Fig. 9:**
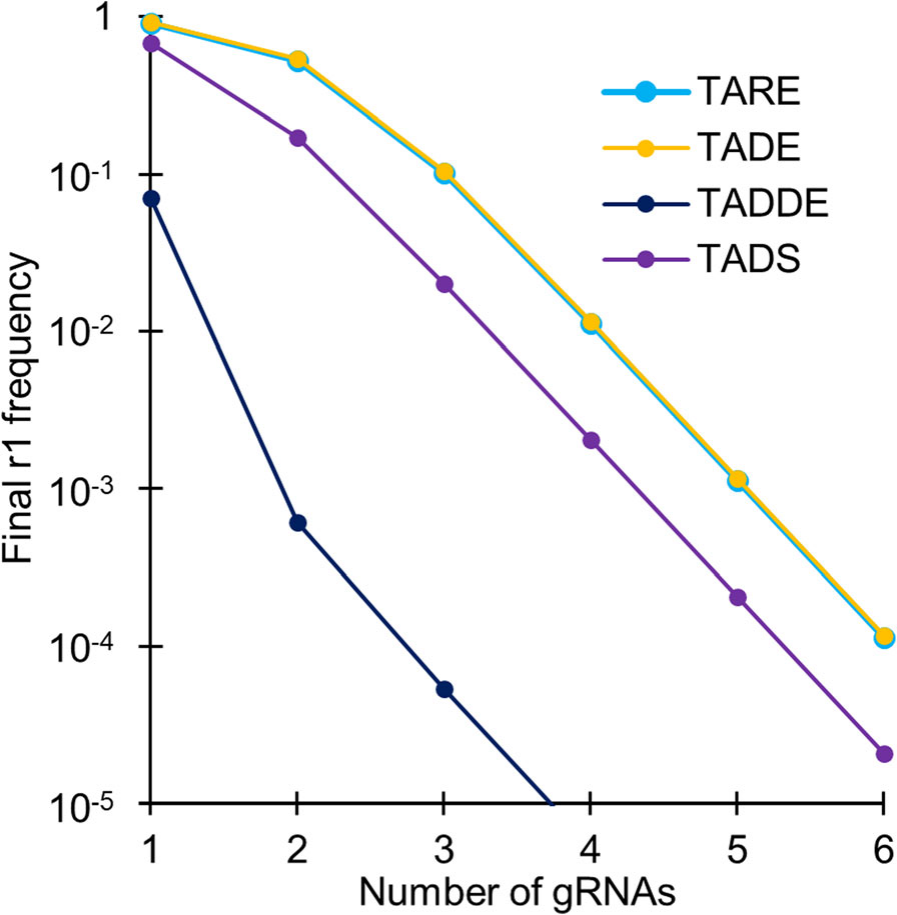
Resistance to TA systems. Analysis was conducted for drive systems with 100% cleavage rates (germline only for TADE) and 95% drive homozygote fitness. Each cleavage event was assumed to result in a functional r1 allele instead of a disrupted target allele with 10% probability. The number of gRNAs was varied, and a resistance allele was considered to be a “complete” r1 allele only if all gRNA cleavage sites possessed r1 sequences. The vertical axis shows the frequency of complete r1 alleles after 100 generations

If a distant-site TADS drive is located on the Y chromosome (with the target on a different chromosome), it will bias inheritance in favor of males (Fig. 8a). This is expected to induce a germline cut rate-dependent genetic load (Fig. 8b) on the population after the drive fixes. According to our deterministic model, this genetic load should be halfway between one and that of a Y-linked X-shredder with a similar X-shredding rate. The overall dynamics of a TADS Y-linked suppression drive should be similar to that of an ideal X-shredder (Figure S1A). Such a drive would have a zero-threshold invasion frequency unless fitness costs are very high and should be highly tolerant of both fitness costs (Fig. 9c) and low germline cut rates (Fig. 9d), though the germline cut rate will still need to induce a sufficient genetic load if complete eradication is desired. A TADS suppression system could also be located on the X chromosome, similarly biasing inheritance in favor of females and thereby eventually inducing population suppression.

### Resistance to TA systems

With a modest degree of multiplexing, TA systems should generate substantially fewer resistance alleles than homing-type drives without sacrificing drive performance, since there is no need for homology-directed repair. To study the rates at which r1 resistance alleles (those which preserve the function of the target gene) are expected to form in such systems, we assumed that cleavage repair at a single site had a 10% probability of forming an r1 allele (instead of a disrupted allele), placing it near the upper end of the likely range of this parameter based on experiments [17–19] (by careful targeting, a significantly lower rate could probably be achieved [26]). The presence of a single disrupted site was considered to be sufficient to render the target gene disrupted, so to form a complete r1 allele, each gRNA target site needed to get an r1 sequence.

In drives with this high r1 formation rate, 100% efficiency, and assuming that drive homozygotes had a relative fitness of 95% compared to wild-type homozygotes, a single gRNA was not sufficient to allow for success of TARE, TADE, TADDE, or TADS same-site modification drives (Fig. 9). Though all drives initially increased in frequency rapidly, the (relatively low) fitness cost of the drive coupled with the high rate of r1 formation resulted in elimination of most drive alleles after 100 generations for TARE, TADE, and TADS. TADDE performed somewhat better, since r1 alleles would not be viable in the presence of a disrupted allele for this drive, while drive alleles would remain viable. Nonetheless, the final frequency of r1 alleles was still high for a scenario with only one gRNA.

As the number of gRNAs is increased, the number of r1 alleles that remain decreases drastically (Fig. 9), indicating that for even very large populations a modest number of gRNAs would likely be sufficient to preclude formation of resistance against the TA drives. Indeed, our calculations may substantially overestimate the number of r1 alleles formed, perhaps even greater than 100-fold. This is not only because we assumed a high proportion of repair resulting in r1 sequences, but also because the possibility for simultaneous cutting was not included in our deterministic model. However, such events should take place quite often, particularly as the number gRNAs increases because even one instance of simultaneous gRNA cleavage would likely cause a large enough deletion to prevent formation of an r1 allele [18, 27]. Additionally, homology-directed repair of drive cleavage using disrupted alleles as a template would likely preclude the formation of r1 alleles, and this was not taken into account in our model. Widely spaced gRNAs could reduce the chance of such events taking place, but also increase the chance of successful disruption of the gene, making optimization of gRNA target spacing a potentially important consideration when designing these drives.

## Discussion

In this study, we have shown that CRISPR-based TA gene drive systems hold strong promise for the development of robust modification or suppression drives. These systems have several major potential advantages over other drive strategies (Table 1).

**Table 1:**
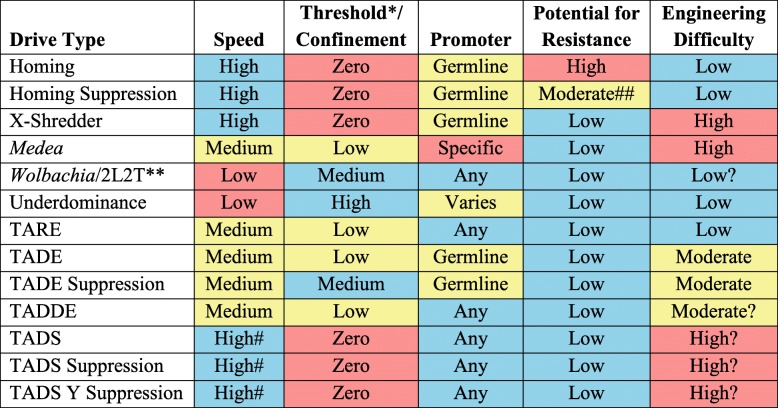
Comparison of drive types. Blue represents high speed, potential for confinement, potential to use additional proter types, potential to avoid resistance, and ease of engineering, while yellow and red represent intermediate and low levels of these attributes

*Thresholds assume a small fitness cost for the drive. Threshold is an indirect measure for the degree of confinement. Zero-threshold drives will potentially spread with even small migration levels, low and medium levels of confinement will constitute regional drives (possibly local for larger fitness costs), and high threshold systems should remain in a local area, if they are able to successfully persist [46]

**A 2-locus 2-toxin-antitoxin (2L2T) underdominance [45, 46, 52] design with two TARE-like alleles [53]

^#^The speed of a TADS drive is reduced if target species females mate multiple times and sperm from different males compete to fertilize eggs

^##^Moderate for formation of resistance, but effects of resistance have a more drastic impact for suppression drives than other types of drives

Perhaps most importantly, TA systems should be far less vulnerable to the formation of resistance alleles than current CRISPR homing drives. Even though multiplexing can somewhat ameliorate the formation of the more critical r1 alleles in homing drives, this typically comes at the cost of reduced drive efficiency due to a variety of factors involved in homology-directed repair [18, 27]. For homing drives designed for population modification, it would also be necessary to target essential genes to remove resistance alleles that disrupt the target function [28], which would open up the possibility for incomplete homology-directed repair to form r1 alleles, possibly at rates that would preclude success of the drive. In contrast, TA drives are not expected to suffer from any efficiency loss upon multiplexing, allowing for effective elimination of r1 alleles given a sufficient number of gRNAs (Fig. 9). The leftmost gRNA need not abut the edge of the recoded region, as in homing drives, making TA systems substantially less vulnerable to incomplete homology-directed repair. Resistance allele formation from undesired homology-directed repair must still be avoided in the design of these drives, but this can usually be accomplished by recoding the area around both sides of the target sites, instead of only the gRNA target sequences themselves [39].

Even if resistance can be avoided in homing drives, they would tend to inactivate any payload gene at a higher baseline rate than TA systems by introducing mutations during homology-directed repair, a substantially more error-prone process than regular DNA replication. Homing drives are also not typically copied in the early embryo by homology-directed repair, and cleavage events that occur in this stage typically result in the formation of resistance alleles for such drives [17–19]. This puts them at a disadvantage compared to TARE, TADDE, and TADS drives, where cleavage events in the embryo would actually benefit the spread of the drive. *Medea* avoids the formation of resistance by the use of RNAi as the drive mechanism, and TA type drives could presumably be engineered similarly to use shRNAs or other RNAi that take effect during early embryo development, though this would somewhat limit the available array of potential gene targets to those with critical function at a developmental stage before the maternal RNAi would be degraded.

Another advantage of TA systems (except for TADS) over “global” homing-type drives is their threshold-dependent invasion dynamics. This would prevent establishment of the drive by occasional long-distance migration, thus confining it to a target region. While zero-threshold drives may be desirable for some applications such as the elimination of a vector-borne disease, regional confinement could often be important for political, economic, or conservation-related reasons [1, 2, 30]. TA systems allow for both regional population modification and suppression, giving scientists and policymakers increased flexibility when considering the deployment of gene drives. Note that TARE and TADE systems, like *Medea*, only have an introduction threshold in the presence of fitness costs. A realistic drive is likely to have a small fitness cost, providing an introduction frequency threshold, though this could possibly also be varied intentionally. Another way to fine-tune the introduction threshold would be to adjust the payload fitness cost, though this would only help with payload confinement, rather than drive confinement (since the payload could be inactivated by mutation). Nevertheless, the small thresholds would render such “regional” drives (drives that only have an introduction threshold if fitness costs are present) still more invasive than underdominance-type “local” drives that have an introduction threshold even without fitness costs, perhaps confining them to larger areas in some situations. Realistic modeling will be needed to determine if a drive can actually be confined in any particular scenario. Because of this uncertainty, we recommend that appropriate biosafety protocols be implemented in experimental research on TA systems to reduce the likelihood of drive spread in the event of an accidental release from the laboratory.

Aside from the drive configurations presented in this manuscript, the same TA principles could potentially be applied to other designs as well. Systems could be developed to utilize additional gRNAs targeting another gene of interest without rescue. This would enable, for example, a same-site TADE drive to be used for population suppression by targeting an essential but haplosufficient female fertility gene, rather than requiring a distant-site drive to be placed inside the fertility gene itself. Such a drive would have identical thresholds to our modeled TADE suppression drive in the ideal case.

Alternative configurations are also possible that would change the dynamics of TA drives [53]. For example, to achieve a greater degree of local confinement (at the costs of greater required release sizes, as is usually the case with such systems), a 2-locus 2-toxin-antidote system [45, 46, 52] could be engineered by using two TARE drives, each providing rescue for the target gene of the other system [53]. Such a system could presumably be engineered quite easily and combined with a tethered homing suppression drive [54], as could other TA systems with an introduction threshold. Note, however, that the germline-only nuclease promoter needed for the tethered homing element may slow down a TARE-based drive due to the lack of embryo activity. Alternatives would be to use TADE drives with germline-only Cas9 expression, different nucleases in the TARE components, or expression of the tethered gRNAs with a germline promoter while retaining a Cas9 promoter that allows for cleavage in both the germline and early embryo. Highly localized suppression could also be obtained with a 2-locus TADE system, with one of the TADE alleles disrupting a sex-specific fertility gene, as in TADE suppression [53]. Indeed, a standard TADE suppression system with a promoter that has high embryo activity could itself be a feasible method for local population suppression, with the level of embryo activity allowing a variable introduction threshold, even without fitness costs [53]. Furthermore, either of these TADE-based methods could be used for population modification if not located in a fertility gene, and a TARE/ClvR drive with a target that is not fully haplosufficient will also have a nonzero introduction threshold even without a fitness cost [40, 53]. Each of these systems should be possible to engineer with current techniques and target genes that are already characterized.

A particularly appealing feature of TA systems lies in the high degree of flexibility they tend to provide in the choice of potential target genes. TARE systems would likely be most efficient when using essential and haplosufficient targets that take effect in the early embryo, to reduce competition among the viable drive-bearing offspring. However, other genes in which disrupted alleles are recessive lethal or sterile (including sex-specific) could also be used. TADE targets should be haplolethal, but some level of haploinsufficiency will usually also be tolerable [53]. On the other hand, TADS targets are highly specific, and this will likely be the limiting factor in the engineering of these systems. Several genes have been found in *Drosophila melanogaster* with post-meiosis I transcription in males [55–57], yet it remains to be seen if a gene can be identified for which expression at this stage is necessary for successful completion of spermatogenesis, thus making it a potential TADS target. It should also be noted that while efforts to design a successful X-shredder system have been stymied by low transgene expression from the Y chromosome, TADS Y-linked suppression systems may not suffer from this issue, since they would only need to cleave a few targets in a single gene, rather than dozens of targets simultaneously across an entire chromosome.

## Conclusion

Overall, our study shows that TA systems can provide flexible and effective mechanisms for a variety of potential gene drive applications. Their feasibility has already been demonstrated experimentally in the case of TARE in *D. melanogaster* for both same-site [39] and distant-site (called ClvR) [40] configurations. Future experiments, simulations, and analytical studies should investigate the feasibility and dynamics of the other TA drives we proposed here and explore how they could be implemented in potential target species such as mosquitoes.

## Methods

### Stochastic simulations

We performed individual-based simulations to study the performance of the different TA gene drive designs. All simulations were implemented in the forward-in-time population genetic simulation software SLiM version 3.2.1 [58]. Our basic model simulates a panmictic population of males and females with discrete, non-overlapping generations. To obtain the individuals of the next generation, each female randomly selects a mate (males can potentially be selected multiple times), with drive-carrying males having a reduced probability of being selected if the drive allele has a fitness cost. The number of offspring generated is then drawn from a binomial distribution with maximum of 50 and *p* = fitness/25, so that a female with fitness = 1, on average, will have two offspring. Fitness is determined by genotype and is multiplied by a density-dependent factor equal to 10/(1 + 9 *N*/*K*), where *N* is the total population size and *K* is the environmental carrying capacity. This density factor was selected to produce logistic dynamics and to smoothly but quickly restore the population to carrying capacity after perturbation, unless a population suppression system produces downward pressure on the population. At low densities, this model produces a maximum 10-fold population growth rate per generation, thus allowing for rapid growth when individuals are not limited by competition.

The next step is to generate offspring. Each offspring randomly receives an allele from each parent. If this allele is wild-type and the parent also had a drive allele, then the wild-type allele is converted to a disrupted allele with a probability equal to the germline cut rate. If the mother had at least one drive allele, then any remaining wild-type alleles in the embryo are converted to disrupted alleles with a probability specified by the embryo cut rate. For the TADS drive, if the offspring received a disrupted allele from its father that would have been carried by a nonviable sperm, the genotype is redrawn. Finally, offspring with nonviable genotypes are removed from the population.

To initialize our simulations, we assumed that drive individuals were first mated with wild-type individuals to produce heterozygous offspring, which were then introduced at a frequency representing 20% of the total population (for the TADS Y-linked suppression drive, all introduced individuals were males with the drive) unless otherwise specified. Several drive performance parameters were fixed at standardized levels inspired by laboratory gene drive mosquitoes [22, 26], unless otherwise specified. These include 99% germline cut rate, 95% embryo cut rate (5% for TADE and TADE suppression drives, which are intolerant of high embryo cut rates—such low embryo cut rates have also been achieved in gene drive mosquitoes [25, 26]), and 95% drive homozygote fitness compared to wild-type individuals. These parameters were then varied in our analyses (individually or in combination) to study how they affect drive dynamics. Each simulation had a starting population of 100,000 individuals (equal to the environmental carrying capacity). The population was allowed to equilibrate for ten generations before adding gene drive individuals, and it was then evaluated over 100 generations.

### Data generation

Simulations were run on the computing cluster of the Department of Computational Biology at Cornell University. Data processing, analyses, and figure preparation were performed in Python and R. All simulations were replicated a total of ten times for each parameter setting, and the results were averaged. SLiM configuration files for the implementation of the simulations and all data are available on GitHub (https://github.com/MesserLab/ToxinAntidoteSystems).

### Deterministic model

Our analyses of the expected behavior of idealized drives shown in Fig. 1 were not inferred from our stochastic simulations. Instead, for these analyses, we used deterministic, discrete generation models for the expected changes in genotype frequencies, specified by recursion equations. This seemed appropriate because we were not so concerned about stochastic dynamics at low population sizes for these analyses. We also verified that allele frequency trajectories in the stochastic simulations converged to those of the deterministic model in the limit of large population sizes.

In the deterministic model, drive/wild-type heterozygotes are initially added to a population of wild-type individuals at a specified introduction frequency. The life cycle in each generation is then modeled as follows: All females select mates proportionally to the male genotype frequencies in the population, further adjusted by the fitness value of each male genotype. Females then generate a number of potential offspring equal to twice their fitness value. Individuals homozygous for a drive allele (or with a drive allele on the Y chromosome) carry a fitness cost as specified. Drive heterozygotes are assumed to have a fitness equal to the square root of the fitness of homozygotes (i.e., we assume multiplicative fitness costs of the drive allele). Several events can take place in the model depending on the particular drive strategy (as specified in further detail for each specific strategy). Offspring with nonviable genotypes are then removed. Genotype frequencies are finally renormalized to produce the population state for the following generation, and the process is evaluated iteratively to obtain the expected allele frequency trajectory in the population over time. To calculate the expected invasion threshold, we systematically varied introduction frequencies to detect the lowest frequency at which the drive was able to invade.

## Supplementary information

**Additional file 1: Figure S1.** Dynamics of TA systems and comparison to other drives. **Figure S2.** Distant-site and X-linked TARE. **Figure S3.** Distant-site TADE. **Figure S4.** Haploinsufficiency in TADE suppression. **Figure S5.** Distant-site TADDE. **Figure S6.** Distant-site TADS.

## Data Availability

SLiM configuration files for the implementation of the simulations and all data are available on GitHub (https://github.com/MesserLab/ToxinAntidoteSystems).
