# Supplementary material for: Performance analysis of novel toxin-antidote CRISPR gene drive systems
Source: BMC Biol. 2020 Mar 12;18:27. doi: 10.1186/s12915-020-0761-2 (PMC7068947; doi:10.1186/s12915-020-0761-2)
Supplement: Supplementary file 1 — Additional file 1: Figure S1. Dynamics of TA systems and comparison to other drives. Figure S2. Distant-site and X-linked TARE. Figure S3. Distant-site TADE. Figure S4. Haploinsufficiency in TADE suppression. Figure S5. Distant-site TADDE. Figure S6. Distant-site TADS. [file 12915_2020_761_MOESM1_ESM.pdf]

SUPPLEMENTAL INFORMATION

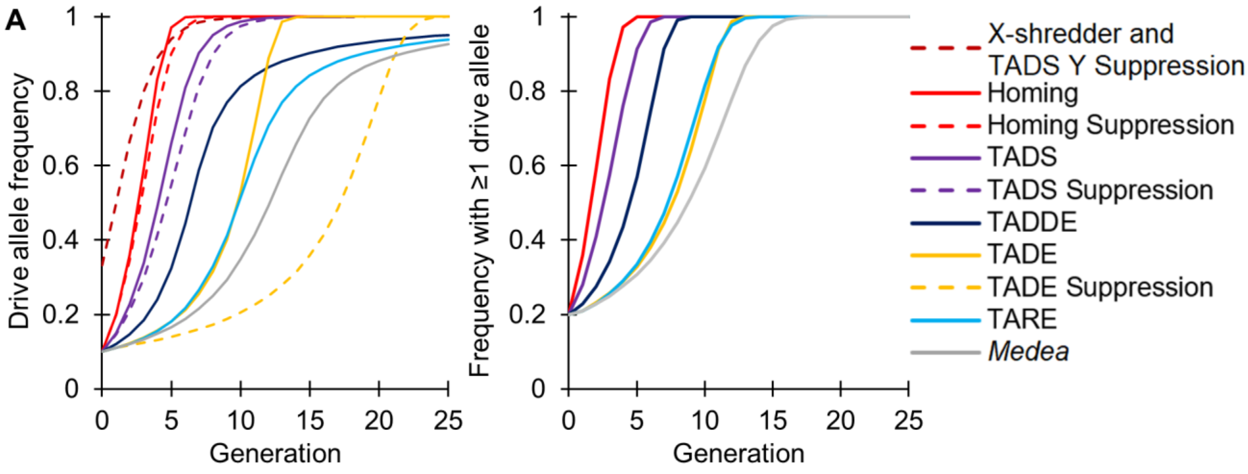

Figure S1. Dynamics of TA systems and comparison to other drives. (**A**) Expected allele frequency trajectories in our deterministic model when drive heterozygotes are released into a wild-type population at 20% starting frequency (10% starting drive allele frequency). The expected trajectories of TADS, TADDE, TADE, and TARE drives are compared with a homing drive, an X-shredder, and a *Medea* system. All drives are assumed to be "ideal" with no fitness cost, 100% drive efficiency, and no resistance evolution. Specifically, drive conversion in the homing drives occurs in both the male and the female germline at 100% efficiency with no embryo cleavage activity. For the suppression version of this drive, we assume that an essential but haplosufficient female fertility gene is targeted, where female homozygotes for a disrupted gene are sterile. For *Medea*, we assume that all offspring of mothers with a *Medea* allele will be nonviable unless they receive a *Medea* allele from either parent. For the X-shredder, we assume that males carrying the Y-linked drive allele shred the X chromosome at 100% efficiency in the germline, and thus, only Y-carrying sperm are transmitted. (**B**) Similar to (A), but instead of showing the total drive allele frequency in the population, the frequency of individuals that carry at least one drive allele are shown (only population modification strategies are included in this figure for easier comparison). Note the more rapid increase for TARE, TADDE, and *Medea* compared with (A).

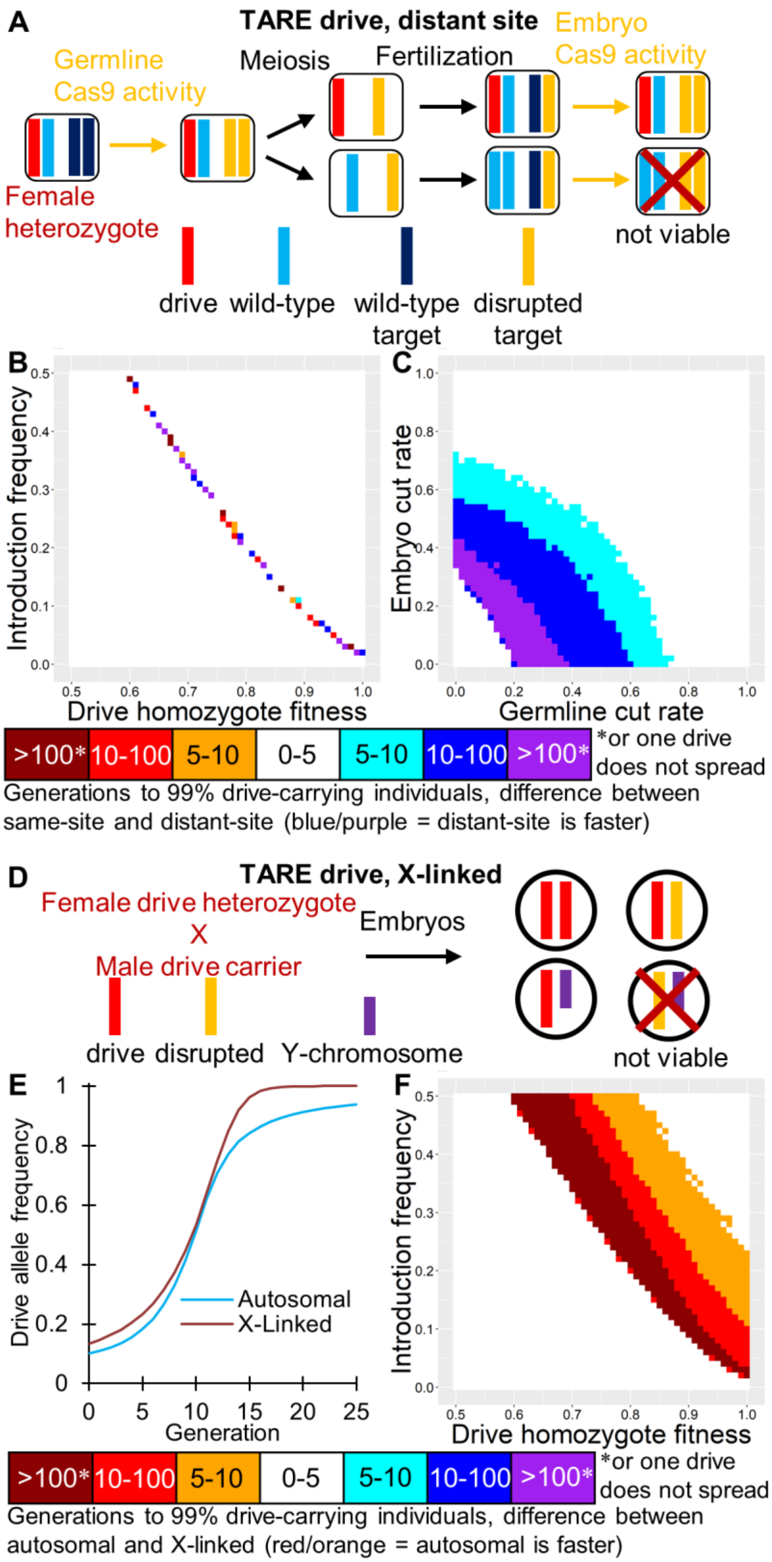

Figure S2. Distant-site and X-linked TARE. (**A**) In the distant-site TARE drive, the target gene is at a separate locus than the drive allele (modeled here as an unlinked site). Individuals

with two disrupted target alleles without any drive alleles are not viable. (**B**) The speed at which the distant-site TARE drive is expected to reach 99% of individuals in the population with varying introduction frequency and drive fitness compared to the same-site drive. (**C**) Same as **B**, but with varying germline and embryo cleavage rate. (**D**) In an X-linked TARE drive, males with only one disrupted copy of the target gene are nonviable. (**E**) An X-linked drive can reach fixation more quickly than and autosomal TARE drive. (**F**) The speed at which the same-site X-linked TARE drive is expected to reach 99% of individuals in the population with varying introduction frequency and drive fitness compared to the autosomal same-site drive.

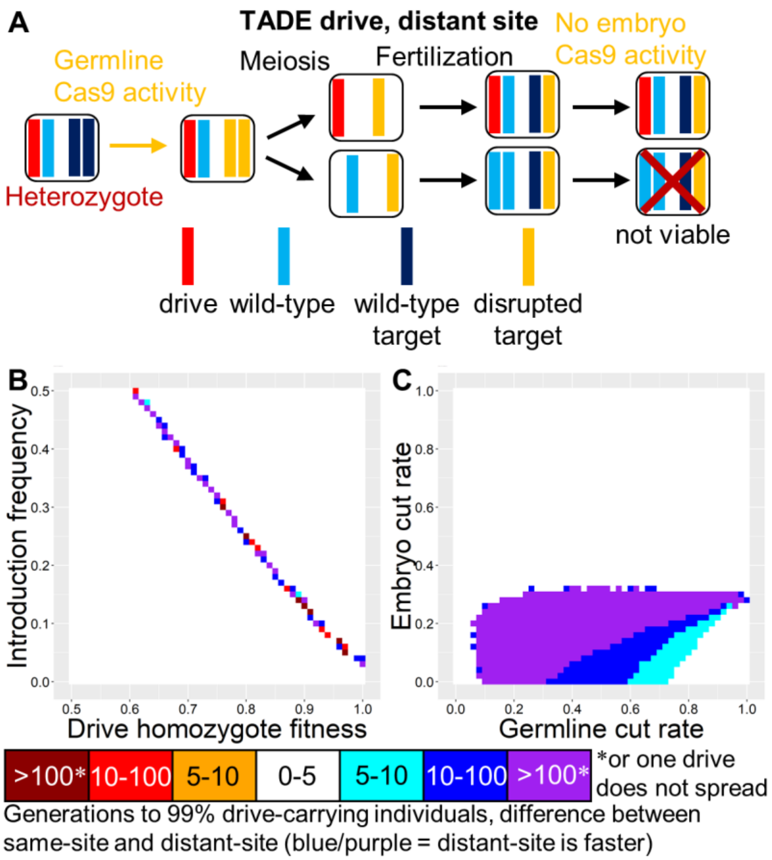

Figure S3. Distant-site TADE. (**A**) In the distant-site TADE drive, the target gene is at a different site than the drive allele (modeled here as an unlinked site). Individuals with fewer than two wild-type target alleles plus drive alleles not viable. (**B**) The speed at which the distant-site TADE drive is expected to reach 99% of individuals in the population with varying introduction frequency and drive fitness compared to the same-site drive. (**C**) Same as **B**, but with varying germline and embryo cleavage rate.

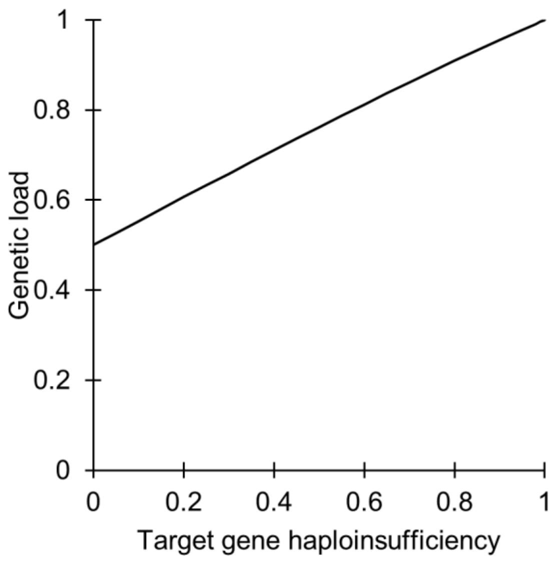

Figure S4. Haploinsufficiency in TADE suppression. If the target allele is not haplolethal but merely haploinsufficient, a TADE suppression drive will impose a genetic load (as calculated with our deterministic model) on the population, which may not cause complete eradication, depending on species and ecological parameters, as well as the degree of haploinsufficiency.

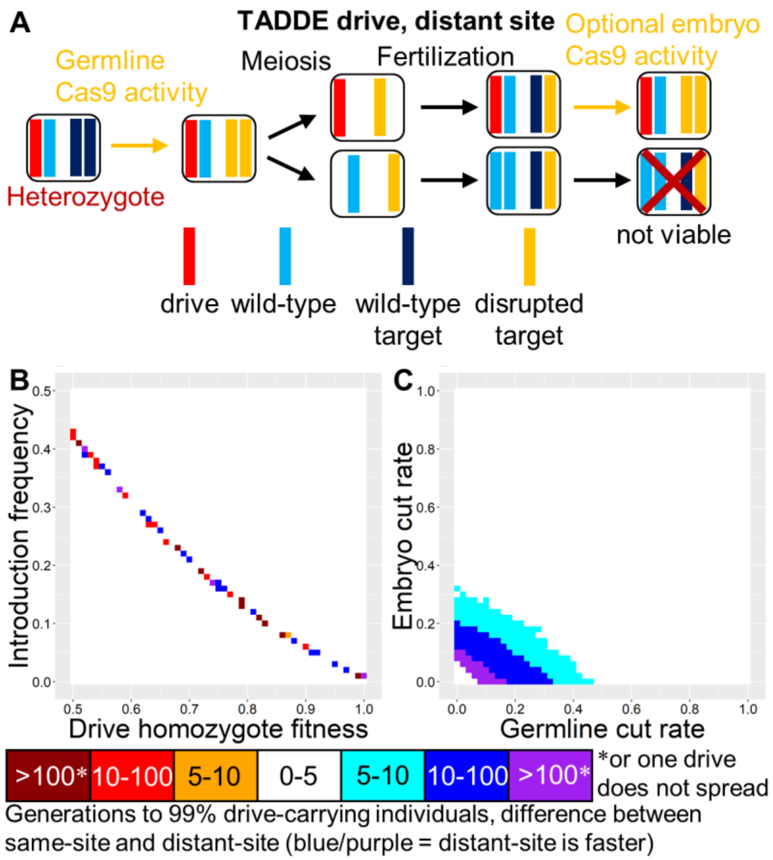

Figure S5. Distant-site TADDE. (**A**) In the distant-site TADDE drive, the target gene is at a different site than the drive allele (modeled here as an unlinked site). Individuals with at least one disrupted target allele are nonviable unless they have at least one drive allele. (**B**) The speed at which the distant-site TADDE drive is expected to reach 99% of individuals in the population with varying introduction frequency and drive fitness compared to the same-site drive. (**C**) Same as **B**, but with varying germline and embryo cleavage rate.

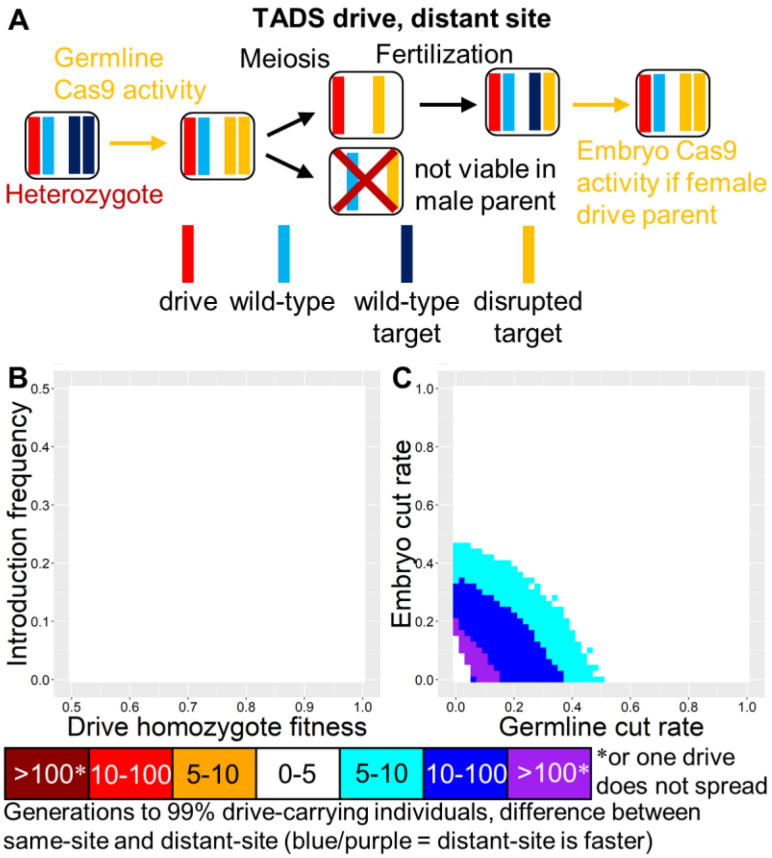

Figure S6. Distant-site TADS. (**A**) In the distant-site TADS drive, the target gene is at a different site than the drive allele (modeled here as an unlinked site). Sperm with a disrupted target allele are not viable unless they also have a drive allele. (**B**) The speed at which the distant-site TADS drive is expected to reach 99% of individuals in the population with varying introduction frequency and drive fitness compared to the same-site drive. (**C**) Same as **B**, but with varying germline and embryo cleavage rate.
